# The Certificates of Family Physicians

**Published:** 1873-09

**Authors:** William C. Wey

**Affiliations:** Elmira, N. Y.


*The Certificates of Family Physicians.* By William C. Wey, M.D., Elmira, N. Y.

An engagement between a physician and his patient, in its mutual obligations, is as binding, morally and legally, as any other implied contract, and the failure of either party to perform makes him liable for the consequences of his dereliction. In this respect the profession of medicine possesses no advantages over the trades or the ordinary commercial relations of society. Estimated by such a standard, medicine, while advanced to the dignity of a profession, is surrounded by and made subservient to the laws and usages which govern the arts and crafts, and is equally amenable to judicial review, discipline and punishment. Unlike the arts and crafts, however, medicine is obedient to a law within itself, which may or may not find expression in a code of carefully-framed rules. Long before a written code of ethics found favor in the profession, which is a very modern suggestion, a sense of personal or individual honor among physicians served to keep inviolate the nature and terms of the engagement or contract between them and their patrons. The force and character of this agreement, though not strengthened by a written code, simply finds amplification in its precepts and declarations.

In a better, in one sense, though not in a more scientific or learned age, when a higher standard of honor prevailed, a written code was not required. Ignorance of professional ethics could not be accepted in extenuation of their violation. In a looser period, with cheapened education, and, as a consequence, diminished honor and responsibility, a code of rules became necessary for the purpose of keeping the ranks in the profession informed and educated up to the standard of accountability current among the wiser and more loyal members of the brotherhood. In these days, as in former days, with or without a written code, a few lead the way, and the many follow or disregard the call, as they are impelled by education, habit, policy, or some other motive.

I am led to consider this subject in connection with the question—"Ought a family physician to grant a certificate in case of application for life insurance?"

I have no hesitation in asserting that it is no part of his duty to furnish information to a life insurance company in respect to the health of individuals who may have placed themselves under his professional care. Not only is it no part of his duty as a medical man, but it is virtually a betrayal of the trust and confidence imposed in unreserved relations between patient and physician.

Even with knowledge that the person to be insured is, and always has been, in such absolute health as to make reply to the questions asked on such an occasion a mere matter of form, and an endorsement of his physical and mental state, like endorsement of his credit or character, it is quite as much a professional act and service as if the physician's statement raised a doubt in respect to the integrity of the applicant's pulmonary or psychological functions.

If it is a friendly office purely, it carries professional significance along with it, thereby violating obligation on the one hand under cover of a personal favor, and communicating valuable information to a life insurance company on the other.

If the certificate is given for a fee, paid indifferently by the party seeking to be insured or by the company, it suggests an imputation that a moneyed compensation may influence the judgment to be rendered. This objection, in view of the paltry sum usually paid by an insurance company, is scarcely worthy of consideration. In the former case, it may be well to observe that an opportunity is offered an unscrupulous applicant and an equally unscrupulous "family physician" to combine, and for a purpose to produce a certificate, which shall reveal a standard of health upon which a policy of insurance will be sure to follow. If a physician, occupying the position of medical examiner for a highly reputable life insurance company, can be found so culpable and criminal as to recommend a consumptive, in the last stages of disease, as a first-class risk, on whose life a policy is issued, it is not difficult to conceive of collusion between an applicant and a family physician, prompted by motives equally offensive and condemning.

It is exceedingly disagreeable to dwell on this feature of the subject, as evidencing loose morals in the profession. An ideal standard of medicine takes no cognizance of such illustrations of baseness. Every-day practical experience with the profession as it is, and not as it should be, or indeed as it would be if raised to an even or uniform basis by education, has forced upon us the unwelcome conviction that, in spite of codes and journals and books and teaching from an endless variety of sources, the *average* men in our ranks are not above suspicion of being governed by selfish and mercenary motives.

The opinion of a reliable family physician, far beyond the recommendation of a medical examiner, carries weight with a life insurance company. Hence the importance of obtaining his approval of a risk. Paradoxical as it may appear, the physician knows the applicant, the corporeal applicant, more intimately than he knows himself. In the undisguised character of patient, his physical, mental and moral attributes have been clearly revealed to his attendant. Nothing has been withheld, simply for the reason that to keep back information would limit the ability of the physician to render prompt and efficient aid and service.

Considering the confidential relations thus engendered and the value of the information acquired by the physician, the usual questions asked in this connection by a life insurance company—"Have you been in the habit of seeing him frequently? Have you given him medical attendance? If so, for what diseases?"—must appear like an attempt harshly to invade the precincts of the sick-room, and cause the medical attendant to betray the interests of those who have implicitly confided in his truth and honor.

The questions above given cover the whole ground of a physician's intimate intercourse with his patient, laying bare his responsible and guilty acts as well as the infirmities for which he is not accountable.

Surely it is not the object of life insurance companies to seek to compromise the office of family physician, or to invite, or for compensation to engage him to do violence to his scruples and convictions. The custom of requiring a family physician's certificate in application for life insurance was established as a matter of business, without considering the nature of his engagement to his patient or the extraordinary demand which it exacted.

It is remarkable that a common professional sentiment did not, long ago, protest against such an attempt to procure information, on the ground, already mentioned, of infraction of ethics, and disregard of individual obligation and propriety. That a more correct estimate of this question is current in the profession I am disposed to believe, from pretty large observation among my colleagues, and from the more general extension of life insurance interests in every city, village and hamlet in the State. The subject is thus brought directly to the attention of medical men, and they are compelled to give it more than usual scrutiny—such scrutiny as embraces the delicate nature of the duties of the family physician in a specific as well as in a more enlarged and comprehensive field.—*Med. Record.*

